# MYcology Stewardship Tool (MyST): a retrospective, descriptive multidisciplinary team review of antifungal prescribing practice in a UK tertiary centre

**DOI:** 10.1093/jacamr/dlag149

**Published:** 2026-07-28

**Authors:** Bee Yean Ng, Louise Dunsmure, Julie Richards, Teig Parsons, Nicola Jones, Maria Tsakok, Samir Agrawal, Andrew Peniket, Monique I Andersson

**Affiliations:** Department of Pharmacy, Oxford University Hospitals NHS Foundation Trust, Oxford, UK; Department of Pharmacy, Oxford University Hospitals NHS Foundation Trust, Oxford, UK; Department of Pharmacy, Oxford University Hospitals NHS Foundation Trust, Oxford, UK; Department of Pharmacy, Oxford University Hospitals NHS Foundation Trust, Oxford, UK; Department of Infectious Disease, Oxford University Hospitals NHS Foundation Trust, Oxford, UK; Department of Radiology, Oxford University Hospitals NHS Foundation Trust, Oxford, UK; Department of Haemato-Oncology, St Bartholomew’s Hospital, and Centre of Immunobiology and Infection, Blizard Institute, Queen Mary University of London, London, UK; Department of Haematology-Oncology, Oxford University Hospitals NHS Foundation Trust, Oxford, UK; Department of Microbiology, Oxford University Hospitals NHS Foundation Trust, Oxford, UK; Nuffield Division of Clinical Laboratory Sciences, Radcliffe Department of Medicine, University of Oxford, Oxford, UK

## Abstract

**Background:**

Antifungal stewardship (AFS) is essential to managing antimicrobial resistance (AMR), yet remains less well established than antibacterial stewardship. This project aimed to support development of an AFS programme at Oxford University Hospitals NHS Foundation Trust (OUHFT) by characterizing antifungal use and identifying priorities for service improvement.

**Methods:**

This retrospective service evaluation reviewed all adult patients prescribed systemic antifungal therapy between 1 November 2021 and 28 February 2022. Data were collected using the MYcology Stewardship Tool (MyST) and included patient demographics, underlying diagnosis, fungal infection investigations, antifungal indication, prescribing details and reason for stopping treatment. Guideline compliance and stewardship appropriateness were reviewed independently by two pharmacists. Categorical variables were summarized using counts and percentages, and continuous variables using medians and interquartile ranges. Results were reviewed at fortnightly AFS project team meetings to identify opportunities for service improvement.

**Results:**

Two hundred patients, comprising 210 patient episodes, were captured in MyST. Median age was 65 years (IQR 56–73), and 61.5% (123/200) were haematology/oncology patients. Among 140 antifungal prophylaxis prescriptions, 91% (128/140) were guideline compliant and 87% (122/140) were stewardship appropriate. Incorrect duration and poor documentation were the main reasons for inappropriateness. Among 165 antifungal treatment prescriptions, 84% (138/165) were guideline compliant and 85% (140/165) were stewardship appropriate. Incorrect dose or dosing frequency were the main reasons for inappropriateness. AFS project team review identified priority areas for improvement, including staff education, patient information resources and improved therapeutic drug monitoring guidance for complex patients.

**Conclusion:**

This project established the foundations for an AFS strategy at OUHFT and identified priorities for further development, including patient information resources, healthcare worker education and therapeutic drug monitoring guidance. Although MyST supported structured data capture and analysis, its use was labour intensive. A dashboard integrated with electronic patient records and capable of providing real-time antifungal prescribing data would facilitate more sustainable, timely and targeted stewardship interventions.

## Introduction

Antimicrobial resistance (AMR) is recognized internationally as a significant and growing threat to global health. In 2019, 1.27 million deaths were estimated to be directly attributable to AMR globally.^1^ AMR is also affecting food security, environmental wellbeing and socio-economic development.^2^ Consequently, the World Health Organization (WHO) endorsed the UN Sustainable Development Cooperation Framework, recognizing the importance of tackling AMR to achieve some of the Sustainable Development Goals.^3^

Antimicrobial stewardship (AMS) is a coordinated healthcare-system-wide approach to promoting and monitoring judicious use of antimicrobial agents. It is an important process to minimize selective pressure and achieve the best clinical outcomes.^4^ However, these benefits have been discussed predominantly in terms of antibiotic use, which has left antifungal stewardship (AFS) largely under-prioritized.^5^

Invasive fungal infections are associated with high morbidity, mortality and they carry a significant financial burden.^5,6,7^ As the number of immunocompromised patients at risk of invasive fungal infection has increased over time, so has the use of antifungal therapy for both treatment and prophylaxis. Increasing use of these agents increases the risk of the evolution of resistance.^8^

There are significant barriers to implementing an AFS programme in comparison to an AMS programme. One of the primary issues is that fungal infection diagnosis is challenging, with limitations in reliable and timely diagnostic test turn around. Consequently, patients may be prescribed empiric antifungals with significant toxicity to avoid treatment delays. The complexity of antifungal pharmacotherapy, such as drug–drug interactions and therapeutic drug monitoring, further supports the need for a multidisciplinary approach comprising a broad range of expertise, including clinicians, pharmacists, radiologists and laboratory diagnostic specialists.^9,10^

A review of compliance with antifungal guidelines in seven European hospitals and one UK tertiary centre showed that adherence to guidelines is low in most studies, with rates of inappropriate antifungal therapy ranging from 25% to 70%.^11^ We sought to establish a formal AFS programme in a UK tertiary NHS trust and we present the data describing antifungal prescribing practice. The data collection was performed using a standardized data capture tool (MyST) and a multidisciplinary team approach to identify opportunities to build a foundation for a robust AFS service and therefore to improve patient care.^5^

## Methods

### Setting

**Figure 1.**
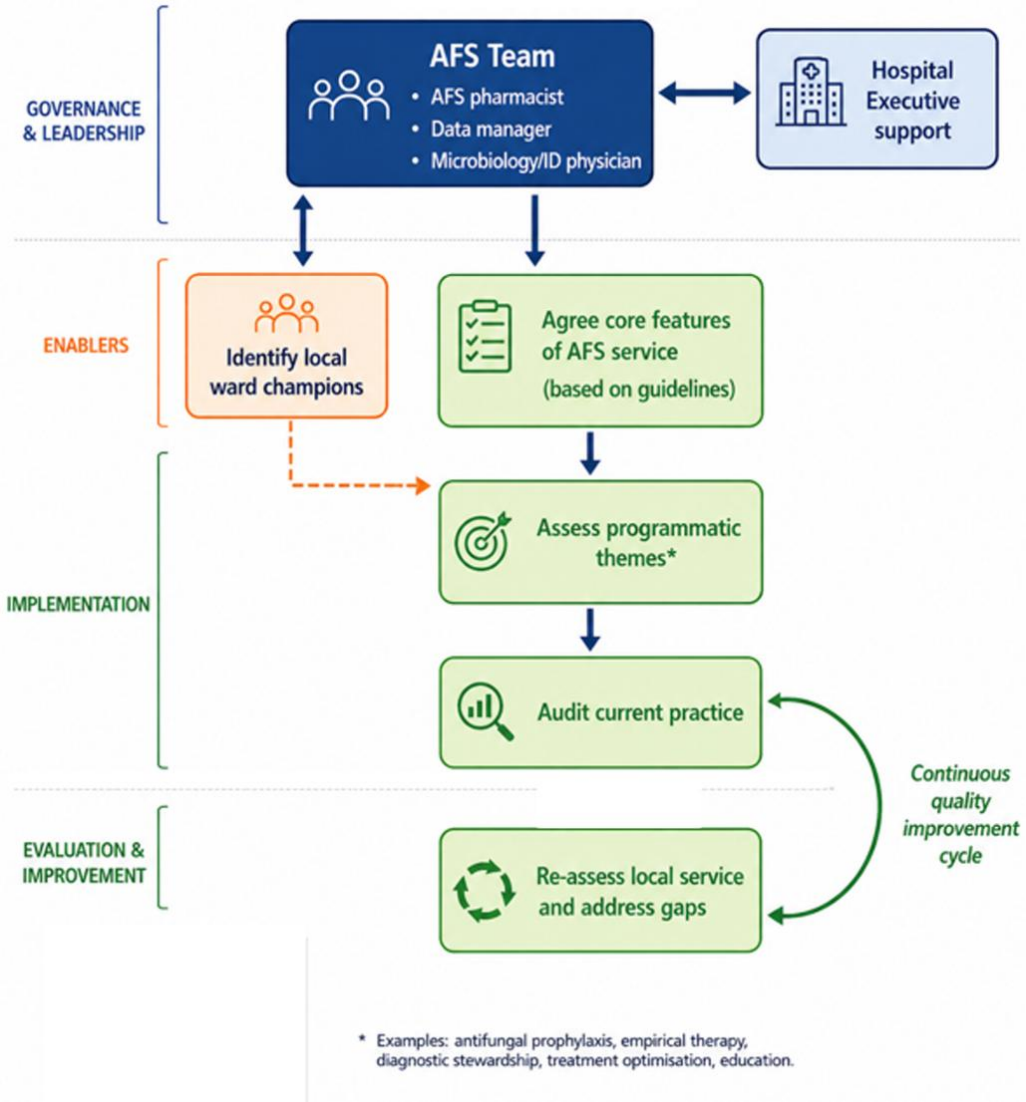
Overview of the AFS Programme. Figure was generated using LLM.

Oxford University Hospitals NHS Foundation Trust (OUHFT) is a large, tertiary teaching trust consisting of four hospital sites in Oxfordshire UK. OUHFT holds 1100 beds and serves ∼1% of the UK population, as well as providing specialist regional referral services (∼3% of UK population) including organ transplant, neurosurgery, haematology/oncology and a bone and joint infection unit. After establishment of a AFS team, securing executive support and identifying ward champions, we established criteria for an AFS programme (Figure 1) according to guidelines.^5^ AFS practice was reviewed using a purpose-built data collection tool—MYcology Stewardship Tool (MyST, available as Supplementary data at *JAC-AMR* online)—as a retrospective, single-centre descriptive service evaluation.

### Data collection

We agreed an ideal model for AFS within the Trust and audited our antifungal use. All adult patients prescribed antifungal therapy in OUHFT from 1 November 2021 to 28 February 2022 were identified using Discern^®^ Analytics, an OUHFT-based data generation programme within the Cerner Millenium Electronic Prescribing and Medicines Administration system.

For these patients, all electronic patient records were reviewed retrospectively. Demographics, diagnosis, indication, investigations, antifungal agent, route and duration, compliance to guideline and appropriateness of antifungal were captured. An ‘episode’ was defined as a new indication for an antifungal prescription. A standard operating procedure for entering patient data was developed to ensure consistency of data collection. Patient data were reviewed to determine the compliance and appropriateness by two pharmacists, to reduce the risk of error. Cases in which there was uncertainty were discussed at the fortnightly multidisciplinary AFS study team meetings.

### Diagnosis and guidelines

Diagnoses of fungal infections were classified according to an adaptation of the European Organization for Research and Treatment of Cancer/Invasive Fungal Infections Cooperative Group and the National Institute of Allergy and Infectious Diseases Mycoses Study Group (EORTC/MSG) criteria combining clinical history, radiology, fungal culture and fungal antigen results.^12^ For non-haematology-oncology patients the diagnosis was based on combination of clinical presentation, radiology, fungal culture and fungal antigen results, interpreted in the context of the level of immunosuppression.

Compliance to guideline was assessed using the locally approved trust antifungal guidelines. These included the prophylaxis and treatment of invasive fungal infections guideline in adult haemato-oncology patient, fungal infection in adult respiratory patients guideline (available on the OUHFT intranet) and the solid organ transplant protocol.^13^ For rare mould infection the European Confederation of Medical Mycology guideline was used.^14^

Stewardship appropriateness was assessed separately considering compliance with the guideline, mycology investigations, including culture and sensitivity, and clinical presentation with consideration of the level of immunosuppression, in line with the Australian Hospital National Antimicrobial Prescribing Survey appropriateness definitions.^15^ Where identified, more than one reason of inappropriateness per prescription was included in the analysis.

Poor documentation was defined as absent or unclear documentation of indication, antifungal choice or intended duration of therapy that was judged by MDT to contribute to inappropriate prescribing or to limit antimicrobial stewardship review and clinical decision making.

All cases in which the classification or the appropriateness or stewardship of antifungal use was not clear were discussed in a fortnightly multidisciplinary team meeting of the AFS team.

### Sample size

A sample size of ∼200 antifungal prescribing episodes was considered sufficient to provide adequate clinical diversity across patient groups, enable meaningful assessment of prescribing practice and evaluate the usability of the data collection tool, as well as identify any AFS gaps.

### Qualitative analysis of reflective discussion

The multidisciplinary AFS team reviewed the results at fortnightly meetings, which prompted reflective discussion to identify potential opportunities to improve the AFS service.

### Ethics approval

The project was registered as a service evaluation and therefore ethical review and approval was not required. It was logged and approved on the Trust Quality Improvement Database.

## Results

Two hundred patients were recruited representing 210 patient episodes. Eight patients were admitted twice, and one patient was admitted three times. 305 discrete antifungal prescriptions were reviewed. No readmissions were judged to be attributable to antifungal prescribing inappropriateness.

### Demographics

The demographics are shown in Table 1. The median age of the cohort was 65 years (IQR 53–73 years), and 65% of patients were male. Ethnicity was not well documented and the results were not included.

**Table 1.** Patient demographics

		Haematology-oncology (*n* = 123)	Non-haematology-oncology (*n* = 77)
Count of patients	Female	39 (31.7%)	32 (41.6%)
	Male	84 (68.3%)	45 (58.4%)
Age (years)	Median age	65.0	70.0
	IQR	53–73	54–81

**Figure 2.**
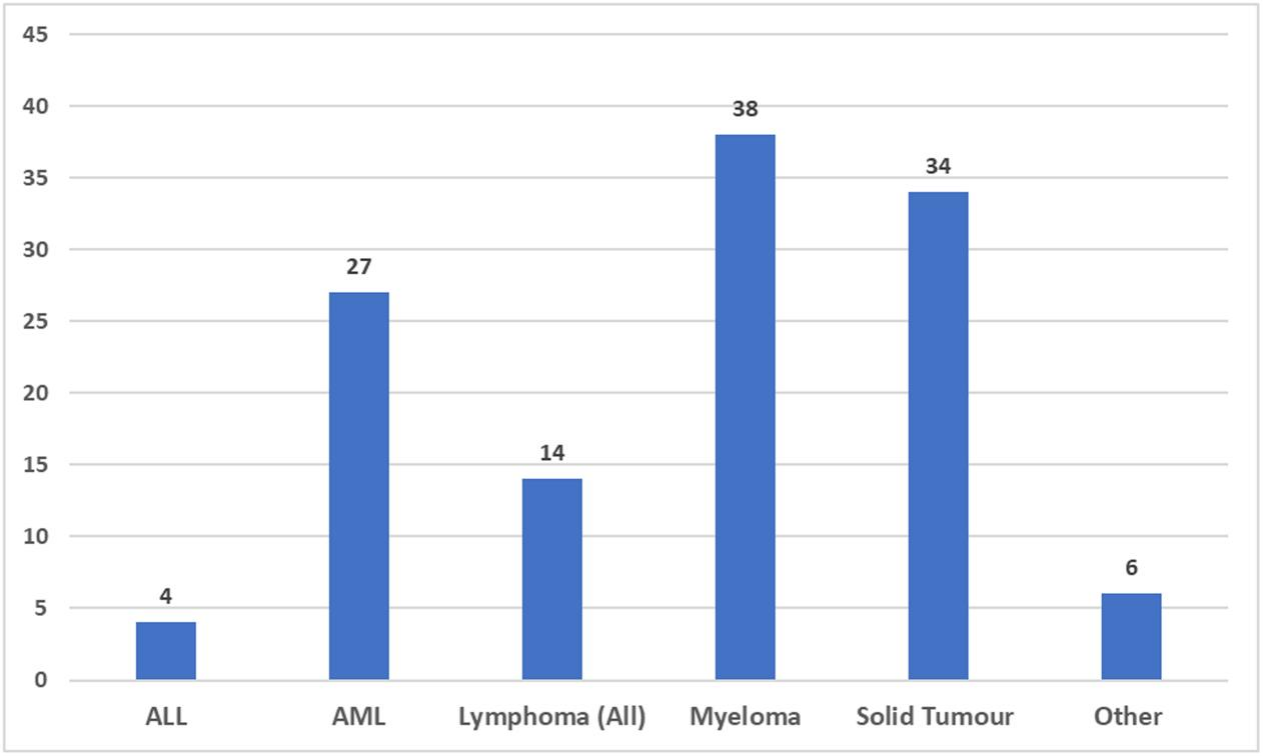
Distribution of haematology-oncology diagnosis. ALL, acute lymphoblastic leukaemia; AML, acute myeloid leukaemia; solid tumour: colorectal, 7; pancreas, 6; oesophagus, 2; lung, 5; prostate, 2; sarcoma, 3; melanoma, 2; oropharyngeal, 2; other solid tumour, 5; other: MDS, 3; plasmacytoma, 2; Waldenström macroglobulinaemia, 1.

Haematology-oncology patients made up 61.5% (123/200) of the reviewed patients. Figure 2 shows the primary diagnosis for the haematology-oncology patients. Other specialities included general internal medicine 19% (38/200), surgery 9.5% (19/200), transplant 5% (10/200) and the ‘other’ 5% (10/200).

### Antifungal use

Eight different antifungal agents were prescribed for patients during this review period: including liposomal amphotericin B, echinocandins and azoles. Of the 305 prescriptions, 140 (46%) were for antifungal prophylaxis and 165 (54%) for treatment Table 2.

**Table 2.** Comparison of frequency of agents used for antifungal prophylaxis versus treatment

Agent	Prophylaxis prescription *n* = 140 (%)	Treatment prescription *n* = 165 (%)
Liposomal amphotericin B	6 (4.3)	16 (9.7)
Fluconazole	72 (51.5)	103 (62.5)
Isavuconazole	0	5 (3)
Itraconazole	1 (0.7)	3 (1.8)
Posaconazole	45 (32.1)	1 (0.6)
Voriconazole	7 (5)	12 (7.3)
Micafungin	9 (6.4)	22 (13.3)
Caspofungin	0	3 (1.8)
**Total**	**140**	**165**

#### Antifungal prophylaxis

**Figure 3.**
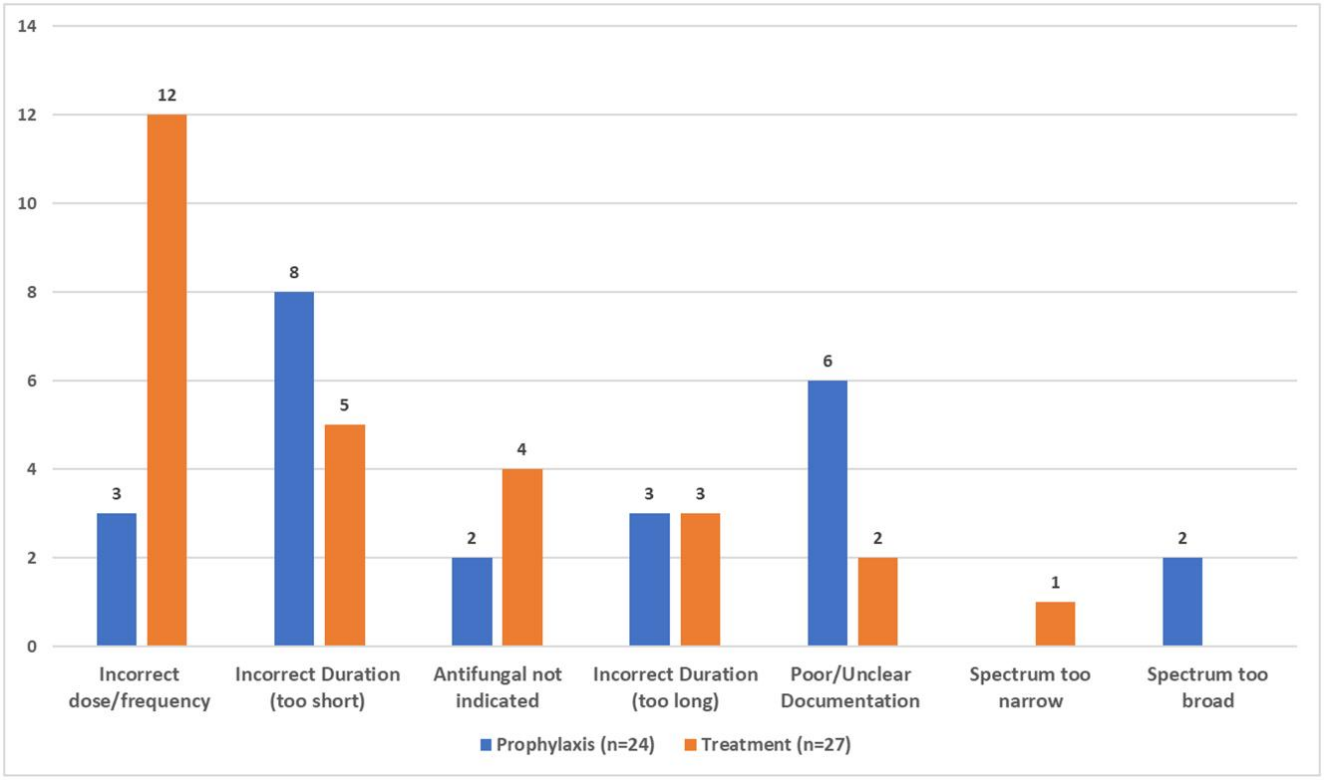
Antifungal prophylaxis and treatment: reasons for stewardship inappropriateness (more than one reason may apply).

In total, there were 140 prescriptions of antifungal prophylaxis with 91% (128/140) compliant with guidelines and 87% (122/140) stewardship appropriate (Figure 3).

According to OUHFT Haematology and Oncology Guideline prophylaxis should be prescribed for the duration of neutropenia until consistent count recovery (neutrophils >1 × 10^9^/L); however, in five patients, antifungal prophylaxis was stopped when neutrophils were still <0.5 × 10^9^/L.

Eighteen of 140 prophylaxis prescriptions (13%) were deemed stewardship inappropriate; across these prescriptions, 24 reasons for inappropriateness were identified, as more than one reason could be assigned per prescription. Incorrect duration (too short for the indication) [33% (8/24)] and poor documentation [25% (6/24)] were the main reasons for stewardship inappropriateness, followed by incorrect dose 13% (3/24), prolonged duration of therapy 13% (3/24), spectrum too broad 8% (2/24) and antifungal not indicated 8% (2/24).

Six prescriptions were assessed as guideline compliant, but stewardship inappropriate. Where the study team were unable to determine the appropriateness due to poor documentation and since the choice of antifungal was as per guideline, they were labelled as guideline compliant but stewardship inappropriate. Of the 140 antifungal prophylaxis prescriptions, 105 stop reasons were documented or collected with the most common reasons being de-escalation, no longer immunosuppressed followed by end of treatment and adverse reaction (Table 3). Ten patients were on antifungal prophylaxis and then required treatment with antifungals. Nine patients were haematology-oncology with one transplant patient (small bowel and pancreas) (Table 4). Only one of these patients had microbiologically confirmed infection on culture.

**Table 3.** Stop reason for antifungal prophylaxis prescriptions

Stop reason (prophylaxis)	*n* = 105
De-escalation	21
Neutrophil recovery/in remission/no longer immunosuppressed	16
End of treatment	13
Adverse reaction/side effect	7
Dose or frequency change	6
Death	7
Escalation	8
Drug interaction	5
Not indicated	3
Unable to take oral medication	3
Allergic reaction	2
Compliance	2
Targeted therapy	2
IV to oral	1
Other	9

**Table 4.** Patients with possible breakthrough infection

Study number	Age	Sex	Diagnosis	Prophylaxis	Diagnosis	Antifungal	Duration (days)
2	56	Female	ALL	Posaconazole	Radiology	liposomal amphotericin B	8
5	45	Male	MDS	Voriconazole	Radiology	liposomal amphotericin B	8
6	51	Female	Burkitt	Posaconazole	Clinical	Fluconazole	6
13	61	Male	AML	Posaconazole	Clinical	Micafungin	12
20	60	Female	AML	Fluconazole	Culture^a^	liposomal amphotericin B/Micafugin/	13 3 days liposomal amphotericin B, 10 days micafungin
57	44	Male	AML	Posaconazole	Clinical	liposomal amphotericin B	3
84	64	Male	AML	Posaconazole	Clinical	Caspofungin	3
121	53	Male	Transplant (SB and pancreas)^b^	liposomal amphotericin B	Clinical	Micafungin	5

^a^Nakaseomyces glabrata.

^b^Small bowel and pancreas.

ALL, acute lymphoblastic leukaemia; AML, acute myeloid leukaemia/

#### Antifungal treatment

In total, there were 165 prescriptions of antifungal treatment with 84% (138/165) compliant with guidelines and 85% (140/165) stewardship appropriate.

Twenty-five of 165 (15%) treatment prescriptions were deemed stewardship inappropriate; across these prescriptions, 27 reasons for inappropriateness were identified.

The incorrect dose or frequency 44% (12/27) were the most common reasons for inappropriateness. Then incorrect duration where the prescribed course was too short for the indication in 19% (5/27) or antifungals were not indicated 15% (4/27), duration was prolonged 11% (3/27), there was unclear documentation 7% (2/27) or the spectrum was too narrow 4% (1/27) (Figure 3).

Of 12 cases of incorrect frequency or dose, 11 involved fluconazole prescriptions. The indication for these prescriptions varied and included superficial *Candida* infection and candidaemia. There were eight prescriptions (seven for fluconazole) that were assessed as guideline compliant, but stewardship inappropriate. This was most often due to missed dosing, poor documentation or clinical reasons such as inappropriate use e.g. treatment of *Candida* spp. colonization.

There were 10 prescriptions (seven for fluconazole) that were not guideline compliant, but stewardship appropriate. Among them were two prescriptions where patients were prescribed isavuconazole as an oral step-down option on microbiology/infectious disease team advice with documented acknowledgement that this was outside OUHFT guidelines. Of the 165 antifungal prophylaxis prescriptions, 140 stop reasons were documented or collected with the most common reasons being end of treatment and de-escalation followed by discharge, dose change and intravenous to oral switch (Table 5).

**Table 5.** Stop reason for antifungal treatment prescriptions

Stop reasons (treatment)	*n* = 140
End of treatment	36
De-escalation	27
Discharge	10
Dose change	9
Resolution of infection	9
IV to oral	9
Escalation	7
Other diagnosis more likely/not indicated	7
Death	7
Targeted therapy	4
Unable to take oral medication	4
Renal impairment	2
Drug interaction	1
High drug level on TDM	1
Other	7

### Fungal infection diagnosis

#### Cultures and fungal antigen testing

There were 30 positive fungal cultures during the observation period. *Candida* spp. were most commonly cultured, with 48% (13/27) identified as *Candida albicans*. Two cases with *Aspergillus* spp. in sputum were identified, and one with *Exophiala* spp. in sputum.

Of 30 positive cultures, resistance testing was performed in 21 cases. High level resistance to one or more agents was seen in seven cases (30%). All cultures of *C. albicans* tested for resistance were fully sensitive, apart from one sample which revealed posaconazole resistance.

Beta-D-glucan (BDG) testing of serum was performed in 22 patients, 77% (17/22) of patients were haematology-oncology patients. The test was reported as detected (>80 pg/ml) in four cases (18%). One patient had fungal aortitis with *Aspergillus* spp. confirmed on histology, one patient with rectal cancer who cultured *C. albicans* from urine, but not from blood, one myelodysplastic syndrome (MDS) patient and one small bowel transplant case had low level positive BDG results.

#### CT scans in patients treated for respiratory fungal infection

Patients who were treated with an antifungal and who had respiratory imaging were reviewed. One patient with a background of lung malignancy cultured *Aspergillus* spp. in sputum. Review of the CT scans showed upper lobe cavitation with likely fungal colonization. One case with background of interstitial lung fibrosis on CT scan cultured *Exophiala* spp., the CT showed changes that were reported as ‘possible’ fungal infection. Two patients without culture or serological results had abnormal chest CT scans with features of fungal infection and were treated with antifungals (one patient with refractory MDS and a solid organ transplant patient).

### Outcomes

The 7-day mortality of the 200 patients recruited to this cohort was 7% (13/200) and total 30-day mortality was 20% (39/200). Thirty-one patients were on antifungal treatment in comparison to nine who were on antifungal prophylaxis, with one patient receiving both treatment and prophylaxis.

The 1- year mortality was 42% (84/200) and at 2 years 50% (100/200) of patients had died. Median age was 71 years and the proportion of males was 60%. Of the 100 patients who died at 2 years, 65% (65/100) were haematology-oncology patients.

## Project group review and reflective discussion

Discussion within the AFS project group identified three priority areas for service development. First, the group identified a need for patient-facing information on antifungal treatment. Second, antifungal therapeutic drug monitoring (TDM) was highlighted as an important way to support prescribing in complex patients, particularly as no clear local TDM guidance was available at the time. Case review identified examples in which TDM may have supported dose optimization and informed ongoing treatment decisions. Third, the group identified a need for targeted AFS education for healthcare workers.

## Discussion

Overall, the guideline compliance and stewardship appropriateness for both antifungal prophylaxis and treatment in OUHFT were >80% (87% and 86% respectively). This is higher than previously published studies, where the proportions were 30%–75% .^11^ This may be due to the availability of a local antifungal prophylaxis and treatment guideline for haematology-oncology and consistent support for AMS from an MDT including haematology-oncology and infection specialists, with access to a radiologist. Despite this, we believe there is room for improvement in our service.

For non-haematology-oncology patients, the principles of decision making were based on the published guidelines on management of fungal infection such as candidiasis, rare mould and aspergillosis.^14,16,17^ However, most of the management plans were individualized, based on advice from the microbiology/infectious disease team. This is consistent with previous studies showing that a successful AFS programme relies heavily on multidisciplinary approach including a specialist in infection, the clinical team and specialist pharmacist.^5,18^

A key areas for improvement identified through this study was the quality of antifungal prescription, including inadequate documentation of indication, especially for patients on antifungal prophylaxis and incorrect dosing of antifungal treatment.

While inappropriate prescribing is multifactorial and influenced by human factors, given the availability of the electronic prescribing system, local guidance and relevant diagnostics the non-compliance and inappropriateness are likely to reflect prescriber knowledge gaps or limited familiarity with local guidance. Consequently, education of healthcare professionals to include prescribing, monitoring and administering of antifungals was an important intervention that addressed gaps identified through this project. Having identified this gap we have developed educational material for healthcare professionals that has been launched at a series of meetings for staff, including clinicians, pharmacy staff and nurses with ongoing programme of education in place. The material included diagnosis of fungal infection, dosing and indications for antifungal therapy.

AFS encompasses the management pathway from diagnosis to optimal delivery of therapy. Patient education is a critical part of this process. Improving understanding of the indication, duration and ‘how to take these medications’ can enhance adherence and optimize absorption, thereby supporting the appropriate and effective use of antifungal agents. We note that two patients in our project stopped antifungal prophylaxis because of reasons related to compliance. At present, there is limited information on the level of public awareness of fungal disease in the UK. Data from the USA indicated that in general public awareness of invasive fungal infection is low.^19^ We have developed an information leaflet for patients, which is used by healthcare professional as part of counselling and patients are provided with a copy if they are discharged on an antifungal.^20^ Future work will explore how to make the provision of information to patients more user friendly. We have also produced antifungal TDM guidelines including information about specific patient groups where obesity, gastrointestinal absorption, toxicity or poor compliance concerns are present, or for those who are on liquid posaconazole, and initiating or stopping a drug that affects azole metabolism.

This project did not record information on potential cost savings, but many studies have demonstrated the cost savings potential of having a comprehensive AFS programme.^21,22,23^ It should be noted that although supplies of generic caspofungin and amphotericin B will inevitably led to a fall in drug costs, the problem of increasing resistance to these agents is inevitable as use increases. This will probably result in greater cost associated with newer antifungal agents with broader therapeutic activity.

The limitations of antifungal diagnostics are evident in this study with only 30 patients with positive fungal culture and only 22 BDG tests performed. The latter has increased significantly since the introduction of in-house BDG testing. The Trust now tests ∼80 patients/month. The diagnosis of fungal infection is challenging and the delays in instituting testing, challenges in the performances of the tests and the delays in results all contribute to increasing antifungal use. Recent guidance has highlighted the importance of timely diagnosis and innovative technologies to improving test performance and increasing access to current technologies will result in improved AFS.^24^

A minority of NHS Trusts have a dedicated AFS programme, and only 43% include AFS within existing antimicrobial stewardship activities.^25^ These findings suggest that AFS remains underdeveloped and is often considered separately from broader AMS structures. However, evidence supports the value of integrating antifungal prescribing within established stewardship processes. In a large Australian study, prescriptions approved through local AMS processes were significantly more likely to be appropriate than those that were not approved (95.9% versus 82.9%, *P* < 0.001).^26^ Specialist facilities managing immunocompromised populations had both higher antifungal use and higher prescribing quality than general acute public hospitals.^26^ Collectively, these data highlight the need to raise the profile of antifungal stewardship in the UK and to embed AFS within routine AMS activity. Positioning AFS as a core component of AMS, rather than as a separate or siloed programme, may reduce perceived barriers to implementation, support wider uptake and strengthen the case for funding and workforce development.

Last, the project was initially based on the assumption that AFS data would be readily available. However, the data extraction, validation and interpretation required greater resources than anticipated. For 210 episodes this project required substantial multidisciplinary input over a 12-month period, including dedicated pharmacist time, a part-time data analyst and ID/microbiology input for data review in a team meeting at least fortnightly.

Electronic patient records are increasingly available in hospitals within the NHS. Real-time auto population of key variables into an AFS platform with a user-friendly dashboard would be an important step forward, and would provide opportunity to inform real-time antifungal decision making and provide a resource for AFS audit.

## Limitations

This review only covered a 4-month period, from November 2021 to February 2022, so differences in prescribing over time will not have been captured. We only collected prescribing data and did not formally measure patient compliance. Fungal infections are relatively rare and the immunocompromised patients in our Trust are prescribed prophylactic antifungals. There were insufficient data to calculate incidence in this cohort.

### Conclusions

The clinical complexity, cost and morbidity of invasive fungal infections, alongside the growing threat of antifungal resistance, support the need for AFS programmes. This study describes antifungal use data and shows that structured review can identify priorities for service improvement. Manual data collection limited efficiency, highlighting the need for real-time dashboards linked to electronic patient records. Future work should consider embedding AFS within existing antibiotic stewardship infrastructure, which may improve sustainability, support wider implementation and strengthen AFS as a core component of AMS.

## Supplementary Material

dlag149_Supplementary_Data

## Supplementary data

The Modified MYcology Stewardship Tool is available as Supplementary data (available as Supplementary data at *JAC-AMR* online).
